# Supplementary material for: Proteomics Analysis of Plasma Membrane Fractions of the Root, Leaf, and Flower of Rice
Source: Int J Mol Sci. 2020 Sep 23;21(19):6988. doi: 10.3390/ijms21196988 (PMC7583858; doi:10.3390/ijms21196988)
Supplement: Supplementary file 1 [file ijms-21-06988-s001.zip › Supplement Table 11.docx]

Table S11. Trypsin-digested fragments of rice heterotrimeric G protein subunits.

1. Gα fragments in the plasma membrane fraction of flower

**Fragment Observed　 　Mr(expt) Mr(calc) 　　Expect Peptide**

**1 397.1951 　　1188.5635 1188.5622 0.0026 SHSLSEAETTK**

**2 366.2199 　　730.4252 730.4225 0.0049 ILQETK**

**3 529.3168 　　1056.6191 1056.6179　 0.000036 LLLLGAGESGK**

**4 769.8976 　　1537.7807 1537.7776　 0.0000042 LLFQTGFDEAELR**

**5 397.2267 　　792.4389 792.4381 　　 0.02 ILYEGAK**

**6 604.7866 　　1207.5587 1207.5568　 0.000014 ELSQVESDSSK**

**7 746.3702 　　1490.7258 1490.7253　 0.0000082 YVISPDNQEIGEK**

**8 388.2016 　　774.3887 774.3872 　 　0.000073 LSDIDGR**

**9 816.9247 　　1631.8349 1631.8307　 0.000006 LWQDPAIQETYLR**

**10 625.0035 　　1871.9886 1871.9854　 0.000021 TNGVVQIQFSPVGENK**

**11 454.236 　　906.4574 906.4559 　 　0.00035 LYDVGGQR**

**12 525.288 　　1048.5614 1048.5593　 0.0082 ELFDWVLK**

**13 541.8168 　　1081.619 1081.6172　 0.000054 TSFILFLNK**

**14 632.8248 　　1263.635 1263.6322　 0.000037 VPLSVCEWFK**

**15 494.7591 　　987.5036 987.5025 　 　0.0083 DYQPIAPGK**

1. Gβ fragments in the plasma membrane fraction of flower

**Fragment Observed Mr(expt) Mr(calc) Expect Peptide**

**1 599.3214 1196.6283 1196.6262 0.0002 HAAATASVNSLR**

**2 833.3918 1664.769 1664.7651 0.0000075 TPVSFNPTDLVCCR**

**3 619.3098 1236.605 1236.6026 0.0000082 VYSLDWTPEK**

**4 616.8058 1231.597 1231.5945 0.000025 NWIVSASQDGR**

**5 636.8705 1271.7264 1271.7238 0.00000018 LIVWNALTSQK**

**6 894.8984 1787.7822 1787.7785 0.00018 GYVSSCQYVPDQETR**

**7 746.3688 2236.0847 2236.0794 0.00022 LITSSGDQTCVLWDVTTGQR**

**8 351.7009 701.3873 701.386 0.013 LWDIR**

**9 486.2325 1455.6756 1455.6743 0.00036 TYHGHEGDINSVK**

**10 586.7361 1171.4577 1171.4564 0.00000011 FGTGSDDGTCR**

**11 396.8764 1187.6074 1187.6047 0.00098 TGHQLQVYSR**

1. **1057.4818 2112.9491 2112.9456 0.000056 ISCLGLSSDGSALCTGSWDK**

(C ) Gγ1 fragments in the immunoprecipitation products using the plasma membrane fraction of etiolated leaf.

**Fragments Observed Mr(expt) Mr(calc) Expect Peptide**

**1 351.2144 700.4142 700.4119 0.00014 IQAELK**

**2 697.342 1392.6694 1392.666 2.20E-07 FLEEELEELDK**

**3 88.9183 1575.822 1575.8178 3.00E-11 VSAALQELMVTAESK**

**4 992.4825 1982.9505 1982.952 2.80E-05 ADPLLPVTTGPACQSWDR**

**5 574.2822 1146.5498 1146.5458 6.50E-05 WFEGPQDLR**

(D ) Gγ2 fragments in the immunoprecipitation products using the plasma membrane fraction of etiolated leaf.

**Fragment Observed Mr(expt) Mr(calc) Expect Peptide**

**1 720.8235 1439.6325 1439.6277 1.00E-08 GEANGEEEQQPPR**

**2 623.9476 1868.8209 1868.8136 2.10E-09 NHLRDDAEEEEEVER**

**3 791.4161 1580.8177 1580.8131 3.20E-06 AARPVSGQQQQQQR**

**4 448.5714 1342.6925 1342.6888 3.20E-06 RRPTDVGGGAAMR**

**5 355.1988 708.383 708.3806 0.00028 SVGYVGK**

**6 351.2204 700.4263 700.4231 3.10E-05 LSAAIAR**

**7 991.0071 1979.9996 1979.9952 4.50E-08 SDPLLPVTIGPENASWER**

**8 677.3413 676.334 676.3333 0.0015 WWASK**
